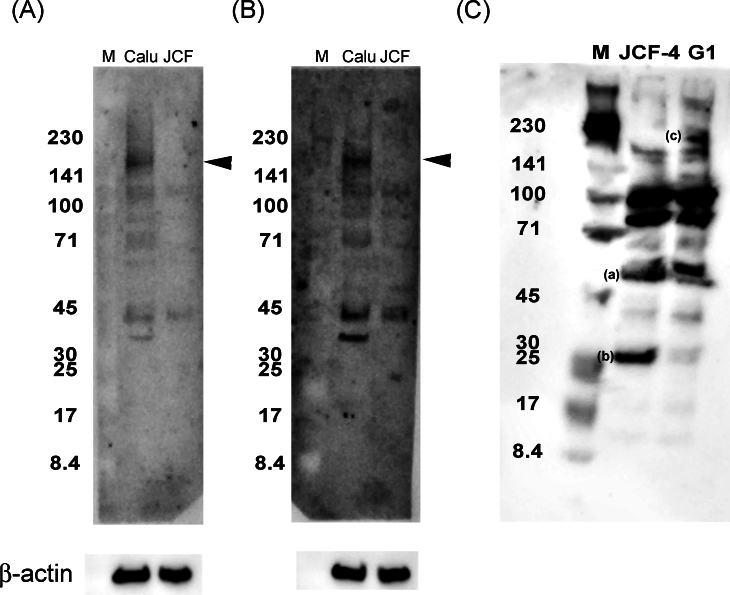# Establishment and characterization of an induced pluripotent stem cell line from a Japanese cystic fibrosis patient with homozygous 1540del10 CFTR mutation

**DOI:** 10.1016/j.gendis.2024.101506

**Published:** 2024-12-25

**Authors:** Hitoshi Okumura, Mikio Hayashi, Hiromi Yamashita, Fumiyuki Hattori

**Affiliations:** a Innovative Regenerative Medicine, Graduate School of Medicine, Kansai Medical University, Osaka 573-1010, Japan; b Department of Physiology, Kansai Medical University, Osaka 573-1010, Japan

Cystic fibrosis (CF) is an autosomal recessive genetic disorder caused by mutations in the CF transmembrane conductance regulator (CFTR) gene.

According to the CF mutation database (http://www.genet.sickkids.on.ca/), among the 2120 known mutations in the CFTR gene, only hundreds have been identified in South and East Asian populations; therefore, the prevalence is 10–200 times lower than Caucasians.^1^ In this study, we identified a homozygous 1540del10 mutation, also referred to as p.Val470GlufsX54, c.1409_1418del, in the CFTR gene of a 13-year-old Japanese male who had been diagnosed as CF by the abnormal elevation of chloride ion concentration in exocrine. This frameshift mutation is harbored in the first nucleotide-binding domain.^2^ We established induced pluripotent stem (iPS) cell lines using the deposited CF patient-derived primary skin fibroblasts (RBC1382, RIKEN BioResourse Research Center). This iPS cell line, named JCF-4, along with wild-type iPS cells (253G1), which had been established from skin fibroblasts of a 36-year-old healthy Caucasian female, were step-by-step stimulated to differentiate into lung epithelial-like cells (LECs) with over 50 days. Immunofluorescent staining confirmed the absence of the plasma membrane localization of CFTR protein only in JCF-4-derived LECs. Whole-cell patch-clamp confirmed defective CFTR-dependent chloride ion currents in JCF-4-derived LECs.

The ethical committee on human rights related to research at Kansai Medical University approved this study involving the genetic characterization of CF patient-derived skin fibroblasts and iPS derivation (No. 2022033). The exosome analysis of the genomic DNA of Japanese CF patient-derived fibroblasts detected the homozygous CFTR sequences with a 10 bp deletion from 1409 to 1418 (Table S1). The polymerase chain reaction across the deletion amplified the single shorter sequence in the case of using cDNA derived from JCF-4 as the template compared with it from 253G1 (Fig. 1A). Furthermore, the Sanger sequencing of cDNA derived from the fibroblasts further confirmed that the CF patient was novel homozygous for the legacy name 1540del10 mutation in the CFTR gene (Fig. 1B), which has been reported as heterogeneous genotypes in two Japanese CF patients.^3^^,^^4^ The mutated CFTR was predicted to have a normal amino acid sequence up to 469 amino acids, be aberrant from 470 to 522 amino acids, and stop codon appearance due to the frameshift. The mutant CFTR protein bears the transmembrane domain (TMD) 1 and a partial nucleotide-binding domain (NBD) 1 (Fig. 1C). The two TMDs are postulated to form the ion pore; therefore, the mutant CFTR protein is supposed to lose the functional ion pore.^2^ The Japanese CF patient-derived iPS cell was developed by an episomal vector-based transient gene expression system. Six genes (human oct3/4, sox7, klf2, L-myc, lin28, and the dominant-negative fragment of p53) and Epstein–Barr virus nuclear antigen 1 were transfected into the patient-derived skin fibroblasts and cultured for up to one month. A total of eight iPS-like cell clones were obtained (Fig. S1A). The karyotype analyses of two clones indicated that one clone had a deletion of one chromosome 11, and another had a normal karyotype. We named the karyotype normal clone JCF-4 (Fig. S1B). Immunohistochemical staining results showed that the JCF-4 cells express the typical pluripotency-related marker proteins, including Nanog, Oct-3/4, SSEA4, Tra1-60, and Tra1-81 (Fig. S1C). To confirm the pluripotency of JCF-4 cells, we conducted a teratoma formation assay with immunodeficient mice following the procedures approved by the ethical committees (No. 24-008) in Kansai Medical University. We found that the developed teratoma contained three-germ layer tissues, including pigmented epithelial, neuronal, cartilage, skeletal muscle, gastrointestinal, and airway epithelial-like tissues, indicating the pluripotent capability of JCF-4 (Fig. S1D).

**Figure 1:**
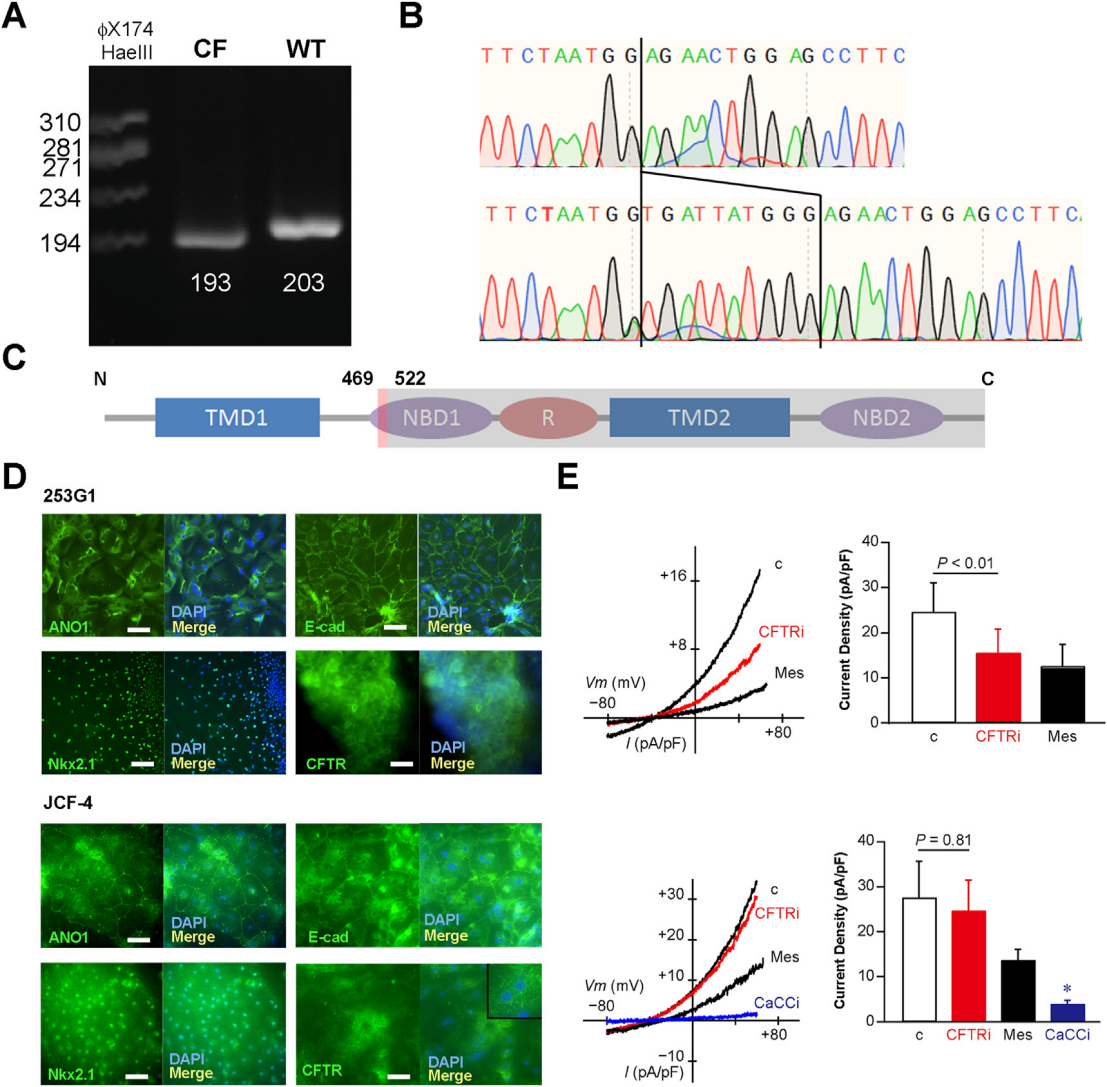
Genotyping of CFTR and characterization of the predicted mutant protein. **(A)** The shorter single band in JCF-4 compared with 253G1 in the amplified cDNA by PCR. The theoretical amplicon lengths are shown. **(B)** The results of Sanger sequencing of cDNA reverse-transcribed from mRNA. Compared with the sequence derived from normal cells, 1409–1418 is missing, and there is no signal derived from another sequence at all (homozygous). **(C)** Predicted amino acid sequence and the corresponding domain. **(D)** Upper panel: 253G1-derived LECs were stained for ANO1, Nkx2.1, E-cadherin, and CFTR. Lower panel: JCF-4-derived LECs were stained for ANO1, Nkx2.1, E-cadherin, and CFTR. Scale bar: 100 μm. The inset indicates the absence of CFTR protein on the plasma membrane and the presence of spotty signals in the cytoplasm. Nuclear DNA is stained by DAPI. **(E)** The traces show current–voltage (I–V) relationships of whole-cell currents recorded from a 253G1-derived (upper row) or a JCF-4-derived (lower row) LEC. The bar graph shows the average current density at 60 mV (253G1, *n* = 7; JCF-4, *n* = 5). Replacement of chloride ion with methanesulfonate (Mes) in bathing solution decreased the outward current. The current was significantly inhibited by 100 μM CaCCinh-A01 (CaCCi) in JCF-4. The application of 20 μM CFTRinh-172 (CFTRi) did not decrease Cl^−^ conductance in JCF-4. ∗*p* < 0.01 or the *p*-value is shown. Error bars: standard errors.

To assess the affection of nonsense-mediated mRNA decay on 1540del10 CFTR, the mRNA expression levels of CFTR in 253G1-and JCF-4-derived LECs were quantitatively measured. As shown in Figure S2, JCF-4 showed significantly decreased expression levels to 23% of 253G1, suggesting that the 1540del10 mutation could trigger moderate nonsense-mediated mRNA decay. Differentiation of iPS cells to LECs was demonstrated according to the stepwise procedures based on the previous report.^5^ The 253G1 iPS and JCF-4 cells changed to an epithelial-like morphology during approximately 30 days in culture. Both 253G1 and JCF-4 formed cystic spheroids spontaneously from differentiation stage 4. However, JCF-4 formed far fewer and smaller cystic spheroids compared with 253G1.

The extracted proteins from the adenocarcinoma cell line Calu-3-, 253G1-, and JCF-4-derived LECs (at differentiation day 53) were applied to western blots and proven by the C- and N-terminal epitope-recognizing antibodies (Fig. S3). The C-terminal epitope-recognizing antibodies detected the theoretical mass of the intact CFTR protein only in Calu-3. The N-terminal epitope-recognizing antibody indicated the unique 30 kDa band in JCF-4, and in turn, over 150 kDa bands in only 253G1. In contrast to 253G1, which showed the theoretically intact mass of CFTR, JCF-4 indicated the smaller molecular weight expected from the amino acid sequence at 522 amino acids, which may suggest the possible degradation.

Immunofluorescent staining for the lung epithelial cell markers, including Nkx2.1, CFTR, ANO1, ZO-1, and E-cadherin, on day 53 showed positive signals in both cell lines. The adhered epithelial-like cells express ANO1, ZO-1, and E-cadherin proteins localizing to the membrane. The cystic spheres derived from 253G1 express the CFTR protein on the plasma membrane; however, JCF-4 did not. Instead of this, the N-terminal epitope-recognizing antibody detected spotty signals in the cytoplasm uniquely in JCF-4-derived LECs (Fig. 1D). The information of all used antibodies is shown in Table S2.

Functional evaluation of CFTR expressed in the 253G1-and JCF-4-derived LECs, which were differentiated for over 50 days, was demonstrated by the whole cell patch-clamp method. All detailed materials and methods can be seen in the online *Supplementary Methods*. The control currents of 253G1-and JCF-4-derived LECs did not show a significant difference. Replacement of chloride ions with methanesulfonate dramatically diminished the currents (Fig. 1E), suggesting steady-state chloride ion secretions in both 253G1 and JCF-4 cells. Only in 253G1, half of the chloride current was significantly inhibited by the CFTR inhibitor, CFTRinh-172, while in JCF-4, the current was not affected (Fig. 1E). The chloride current observed in JCF-4 was completely inhibited by the calcium-dependent channel inhibitor, CaCCinh-A01, suggesting that JCF-4-derived LECs secrete chloride ion mainly by calcium-dependent channels (Fig. 1E). From the above results, we concluded that JCF-4, having homozygous 1540del10 mutations, does not express any functional CFTR protein.

The most common CFTR mutation, F508del, in Caucasians, leads to protein folding and trafficking defects; consequently, corrector drugs have been developed to address these issues. One of the hopeful therapies for rare mutations might be genetic therapies.

## Cell availability

JCF-4 will be available from RIKEN BioResourse Research Center, Ibaraki, Japan.

## Funding

This study was supported by a grant from JSPS KAKENHI (No. 24K11354).

## CRediT authorship contribution statement

**Hitoshi Okumura:** Writing – original draft, Investigation, Data curation. **Mikio Hayashi:** Writing – review & editing, Investigation. **Hiromi Yamashita:** Writing – review & editing, Validation, Investigation, Funding acquisition. **Fumiyuki Hattori:** Writing – review & editing, Writing – original draft, Supervision, Investigation, Conceptualization.

## Conflict of interests

The authors declared no conflict of interests.

## Figures and Tables

**figs1:**
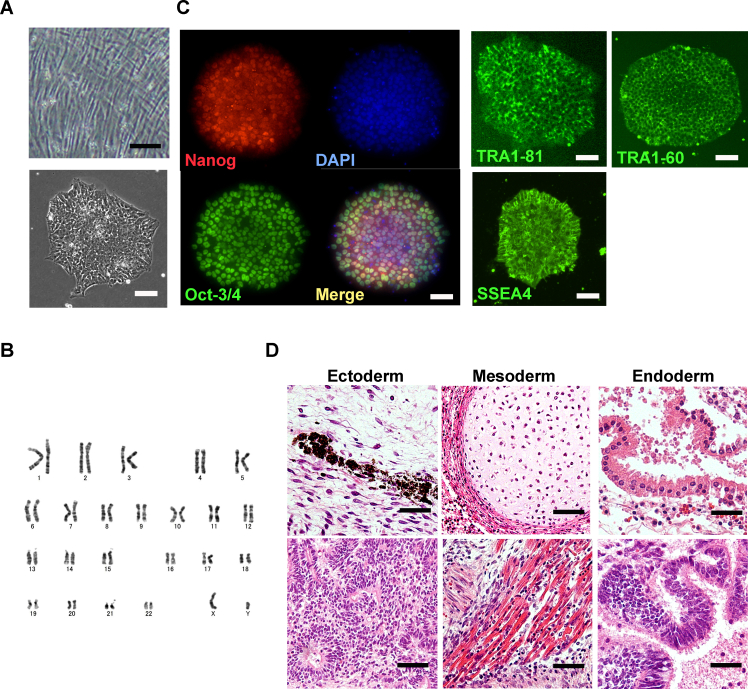


**figs2:**
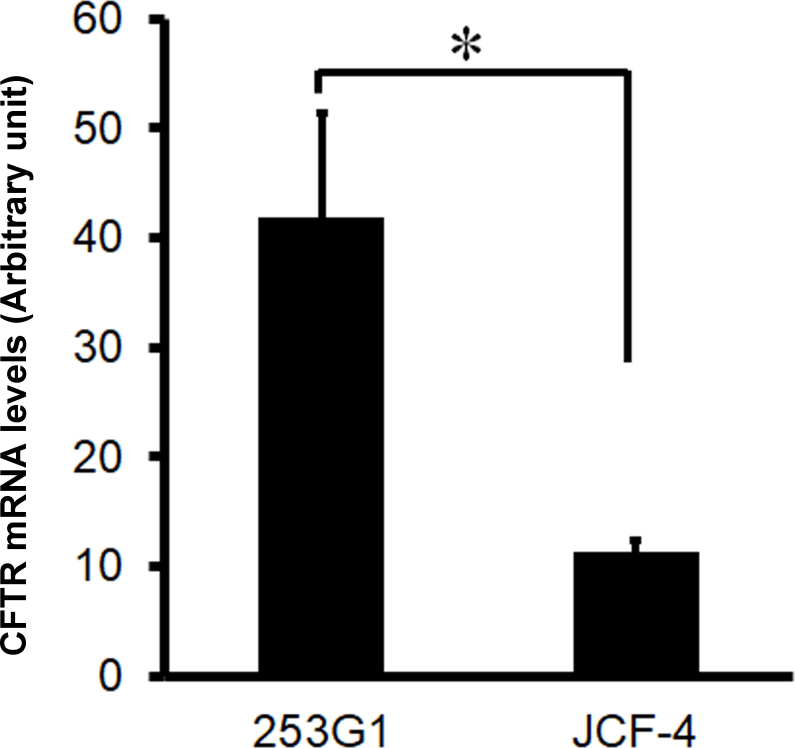


**figs3:**