# Supplementary material for: Establishment and characterization of an induced pluripotent stem cell line from a Japanese cystic fibrosis patient with homozygous 1540del10 CFTR mutation
Source: Genes Dis. 2024 Dec 25;12(5):101506. doi: 10.1016/j.gendis.2024.101506 (PMC12142528; doi:10.1016/j.gendis.2024.101506)
Supplement: Multimedia component 1 [file mmc1.docx]

**Appendix**

**Supplementary Materials and methods**

Animals

NOD-SCID mice (CLEA Japan, Inc., Tokyo, Japan) were used for cell transplantation and histological analyses.

Exome Analysis

Exome sequencing was performed by GeneBay Inc. (Yokohama, Japan). To prepare paired-end libraries, the genomic DNA was fragmented, and their exons were enriched by the SureSelect Human All Exon V6 kit (Agilent Technologies, CA, USA). Sequencing was performed using the Illumina NovaSeq 6000 system. The obtained sequences were mapped onto the human reference genome GRCh37 (hg19) using BWA v0.7.17. Their single-nucleotide polymorphisms, insertions, and deletions were detected using the Genome Analysis Toolkit (GATK, v4.2.6.1).

Sanger Sequencing

The wild-type iPS cells (253G1) ^1^ were obtained from RIKEN BioResourse Research Center. Each 1 μg of the extracted total RNA from the undifferentiated iPSC cell (253G1 and JCF-4) was reverse transcribed using SuperScript® Reverse Transcriptase (Thermo Fisher Scientific, Waltham, MA, USA) with an oligo(dT)20 primer. Each cDNA was applied to the sequencing reaction with the primer　TCAAGATAGAAAGAGGACAGTTGTTG using Bigdye terminator v3.1 (Thermo Fisher Scientific) following the manufacturers’ instructions. The sequence was determined by 3500 genetic analyzers (Thermo Fisher Scientific).

Polymerase chain reaction (PCR) and electrophoresis

The primer pair (1454-GCAGGCAAGACTTCACTTCT-1473 and 1636-TCCTCTTCTAGTTGGCATGCT-1656) was designed by Primer3 on-line software, which theoretically produces 203 bp and 193 bp products, respectively, from wild-type and 1540del10 CFTR. A polymerase chain reaction was performed using KOD Plus (TOYOBO Co. Ltd., Osaka, Japan) with 30 cycle reactions. The agarose gel electrophoresis was performed with 2.5% agar (NIPPON GENE CO., LTD., Tokyo, Japan) in Tris-borate-EDTA buffer (Promega Corporation).

Generation of a human induced pluripotent stem cell

The genetically uncharacterized cystic fibrosis patient derived skin fibroblasts (RBC1382) were obtained from RIKEN BioResourse Research Center, Ibaraki, Japan. The fibroblasts were expanded with MEM-alpha supplemented with 10% heat-inactivated fetal bovine serum. The grown cells were recovered by 0.25% trypsin and 1 mM EDTA in phosphate buffered saline (PBS) (Nacalai Tesque Inc., Kyoto, Japan). To induce iPS generation, the gene sets of human Oct-3/4 (pCE-hOCT3/4), Sox-2 and Klf-4 (pCE-hSK), L-Myc and Lin-28 (pCE-hUL), mouse p53 dominant negative fragment (pCE-mp53DD) and nuclear antigen Epstein-Barr Virus (EBNA1, pCXB-EBNA1) carrying vectors ^2^ were obtained from Addgene MA, USA, and transfected by Fugene-6 transfection Reagent (Promega Corporation WI, USA) to 2 x 10^5^ cells following the manufacturers’ instructions ^2,3^. The gene-transduced cells were seeded on a 10 cm plastic dish (Corning Inc., NY, USA) coated with 0.5 μg/cm2 iMatrix511 Silk (Nippi Inc., Tokyo, Japan) and grown with StemFit AK02N (Ajinomoto, Tokyo, Japan) as the culture medium supplemented with 10 μM Rho kinase inhibitor, Y-27632 (Selleck Biotech, Kanagawa, Japan). The medium was changed once a day to three days, optimized in accordance with the cell density. The emerged colonies with the typical appearance of pluripotent stem cells were picked up manually and separately cultured. Eight independent clones that maintained the typical morphologies of pluripotent stem cells during a few passages were obtained.

Karyotype analysis

Karyotype analysis of hiPSCs was conducted by SRL, Inc. Tokyo, Japan.

Teratoma Formation

Freshly obtained undifferentiated JCF-4 cells detached from the culture dish and dispersed into small clumps were mixed with Matrigel (Corning Inc., NY, USA) in a 1:1 volume ratio. Twenty microliters of the cell suspension solution were aspirated into a 29G syringe, and then injected into the testis of the deeply anesthetized NOD-SCID mouse. Two months later, the mouse was sacrificed, and teratoma was obtained from the intraperitoneal cavity. The produced teratoma was fixed with a 4% para-formaldehyde solution (Fujifilm Wako Chemical Inc., Osaka, Japan) and embedded in paraffin. Five micromater-thick sections were stained with hematoxylin and eosin (HE).

Quantitative PCR

To assess the possible effect of nonsense-mediated mRNA decay (NMD) ^4,5^ on 1540del10 CFTR, the mRNA expression levels of CFTR in 253G1- and JCF-4-derived LECs were quantitatively measured. Each 100 ng of the extracted total RNA from the 253G1- and JCF-4-derived LECs was reverse transcribed using SuperScript® Reverse Transcriptase (Thermo Fisher Scientific) with an oligo(dT)20 primer. Each cDNA was applied to the quantitative PCR with the primer pair for CFTR (2581-GGAGAGCATACCAGCAGTGACT-2602 and 2705-TTCCAAGGAGCCACAGCACAAC-2726), and for ribosomal protein S18 (RPS18, 5’ GCGGCGGAAAATAGCCTTTG and 5’ GATCACACGTTCCACCTCATC) using GeneAce SYBR™ qPCR Mix II (Nippon Gene) and quantitative PCR system, Rotor gene 2 (QIAGEN K.K., Tokyo, Japan). Serial dilutions of one of the samples were amplified for quantification. Each CFTR expression level was divided by RPS18 expression level as the internal control.

Maintenance and differentiation of human iPS cells

The human iPSCs were differentiated into lung epithelial-like cells following the previous report ^6^. Undifferentiated human iPSCs were maintained on plastic dishes (Corning Inc., NY, USA) coated with 0.5 μg/cm2 iMatrix511 Silk (Nippi Inc., Tokyo, Japan) using StemFit AK02N (Ajinomoto, Tokyo, Japan) as the culture medium. We performed the differentiation of LECs referring to the previous publication^7^. The dispersed cells were seeded at a density of 15,000 cells/cm2 with 10 μM Y-27632. One day later, the medium was changed to the same medium without Y-27632. Passage of the cells was performed every three to four days. The cells were detached from the culture dish by treatment with the TrypLE express enzyme (Thermo Fisher Scientific) supplemented with 10 μM Y-27632 for 20 min at 37 °C. For definitive endoderm differentiation, on Day 0, the dispersed 1.0 x106 iPS cells were seeded in a 6-well plate coated with 1/100 diluted Matrigel with Ak02N containing 10 μM of Y-27632. On Day 1 (Stage1), RPMI1640 medium 1/100 B27 supplement (RB medium) containing 100 ng/ml of human activin A (Nacali Tesque) and 50 ng/mL of bone morphologic protein 4 (BMP-4, Peprotech, Thermo Fisher Scientific). On Day 2, the medium was changed to RB medium supplemented with 100 ng/ml of human activin A. On Day 3, RB medium containing 50 ng/ml of human activin A. For anterior superior visceral endoderm induction, on Day 6, the medium was changed to RB medium containing 500 ng/mL basic fibroblast growth factor (FGF2, Nacalai Tesque) , 50 ng/mL sonic hedgehog (SHH, Peprotech, Thermo Fisher Scientific), 2 μM Dorsomorphin (Cayman Chemical MI, USA), and 10 μM SB431542 (Tokyo Chemical Industry Co., Ltd., Tokyo, Japan). For lung epithelial stem cell induction (Stage2), on Day 10, the medium was changed to IF medium consisting of IMDM (Fujifilm Wako) mixed with F12 (75:25, vol./vol., Merck KGaA, Darmstadt, Germany) containing 1/200 N2 supplement, 1% B27 supplement, Glutamax (Thermo Fisher Scientific), 0.15 mM monothiol glycerol (Merck), 0.05% BSA (Merck), 50 μg/mL ascorbic acid 2-phosphate (Merck) containing 10 ng/mL FGF7 (Qkine, Cambridge, UK), 50 ng/ml FGF10 (Peprotech, Thermo Fisher Scientific), 10 ng/mL BMP-4, 3 μM CHIR99021 (Cayman Chemical), 100 nM ATRA (Merck), 10 μM Y-27632. In IMDM/F12 (75:25), 0.5% N2 supplement-A, 1% B27 supplement, Glutamax, 0.15 mM MTG, 0.05% BSA, and 50 μg/mL ascorbic acid. For proximal lung epithelial progenitor cell induction (Stage3), on Day 16, the medium was changed to IF medium supplemented with 50 ng/mL FGF7, 50 ng/mL FGF10, and 10 ng/mL FGF18 (Peprotech, Thermo Fisher Scientific). For differentiation of lung epithelial-like cells (Stage4), on Day 21, the medium was changed to PneumaCult-Ex plus (Stem cell technologies, Vancouver, Canada), 1 μM forskolin (Tokyo Chemical Industry), 10 ng/mL FGF18, 50 ng/mL FGF7, and 50 ng/mL FGF10. Afterward, the medium was changed every three to four days till differentiation day 50 to 60.

Western blot

LECs derived from JCF-4, 253G1 iPS cells, and human adenocarcinoma-derived cell line, Calu3 cells (ATCC, HTB-55) possessing the confirmed expression of CFTR protein ^8^, were washed once with PBS(-) and lysed using RIPA buffer (Nacalai Tesque Inc.) with a protease inhibitor cocktail (#08714, Nacalai Tesque Inc.). The proteins were electrophoresed by sodium dodecyl sulfate-polyacrylamide gel (4 to 12% of Bolt™ Bis-Tris) and a 1.0 mm Mini Protein Gels system and transferred onto a polyvinylidene fluoride membrane (#1214726, GVS, S.p.A. Rome, Italy). The membrane was immersed in blocking one for 30 minutes at 25 °C with gently spaking. The 1st antibodies and 2nd antibodies used are listed in Supplementary Table 1. The luminescent signal was obtained using horseradish peroxidase substrate (Amersham™ ECL™ Prime, Cytiva, Tokyo, Japan) and captured using Fusion Solo-S (Vilber, Collégien, France). The beta-Actin as the internal control was detected using the re-probed membrane treated with stripping solution (Fuji Film Wako Chemical Inc.).

Immunofluorescent staining

To confirm successful differentiation of LECs, we immunohistochemically detected various proteins as shown in the previous report ^6^. The cells were fixed in 4% paraformaldehyde for 20 minutes at 25 °C. They were then washed twice with Tris-buffered saline containing 0.2% Tween-20 (TBS-T) and incubated with 0.1% Triton-X100 containing TBS-T for 10 min at 25 °C. Next, they were treated with a blocking solution (Nacalai Tesque) for 30 minutes at 25 °C. The first antibody-containing blocking agent was applied and incubated overnight at 4 °C with paraffin sealing to prevent evaporation. The cells were then washed three times with TBS-T and immersed in the second antibody-containing blocking agent for 1 hour at 25 °C. After washing three times, fluorescent signals were observed using a fluorescence microscope (Eclipse Ti2, Nikon Instruments, Tokyo, Japan) controlled by the equipped software (NIS-Elements, Nikon Instruments). The primary and secondary antibodies used are listed in Supplementary Table 2. Nuclear DNA was stained by 4',6-diamidino-2-phenylindole (DAPI) (Thermo Fisher Scientific).

Whole cell patch-clamp

Standard patch-clamp techniques were used ^9^. Patch pipettes were made by pulling the capillaries of hard borosilicate glass (G-1.5; Narishige). The pipette solution consisted of 100 mM Cs-gluconate, 10 mM CsCl, 1 mM MgCl_2_, 10 mM EGTA, 10 mM glucose, 2 mM ATP-Mg, 1 mM cAMP, 10 mM HEPES/CsOH, pH 7.4. The cells were perfused with a N-methyl-D-glucamine (NMDG)-Cl bath solution that contained 150 mM NMDG-Cl, 1 mM CaCl_2_, 1 mM MgCl_2_, 5 HEPES/NMDG, pH 7.4. To test the anion selectivity, chloride in the control solution was replaced with methanesulfonate. To evaluate the contributions of CFTR and calcium-induced calcium channel (CaCC) in outward current, 20 μM chemical CFTR inhibitors, CFTRinh-172 (Santa Cruz Biotechnology, TX, USA), and 100 μM CaCCinh-A012 (Cayman Chemical, MI, USA), were applied. The current was recorded in the whole-cell configuration using the EPC 800 patch-clamp amplifier (HEKA Instruments Inc., MA, USA). The amplifier was driven by Clampex 9 (Molecular Devices, LLC., San Jose, CA, USA) in order to allow the delivery of a voltage-ramp protocol with concomitant digitization of the current. The membrane potential was generally held at 0 mV, and the command voltage was varied from +80 to −80 mV over a duration of 800 ms every 10 s.

Statistical analysis

Statistical analyses were performed using EZR software (Jichi Medical University, Japan) ^10^. For comparisons with control, significant differences were determined using one-way analysis of variance (one-way ANOVA), followed by post hoc testing using the Dunnett test vs. control. For comparisons between two samples, a Student's t-test was performed. Statistical significance was set at p < 0.05.

**Supplementary Figure legends**

Supplementary Figure 1, Generation of Japanese CF patient-derived iPS cells.

(A) The upper picture is of Japanese CF patient-derived skin fibroblasts. The lower picture is Japanese CF patient-derived iPS cell clone No.4 (JCF-4). (B) Karyotype analysis of JCF-4. (C) HE staining of the teratoma. Scale bar: 200 μm. (D) The left panel indicates immunofluorescent staining of JCF-4 for Nanog and Oct-3/4. The right panel indicates immunofluorescent staining for TRA1-81, TRA1-60, and SSEA4. Scale bar: 100 μm. Nuclear DNA is stained by DAPI.

Supplementary Figure 2, Comparison of CFTR mRNA levels between 253G1 and JCF-4 by quantitative PCR.

The quantitative PCR analyses for CFTR mRNA levels using cDNA samples from 253G1- (N = 3) and JCF-4-derived LECs (N = 3). *: p < 0.05. The error bars: standard deviations.

Supplementary Figure 3, Western blot of LEC-differentiated (Day 53) proteins derived from JCF-4.

(A, B) The bands detected by C-terminal epitope recognizing antibodies are uniquely seen approximately on the theoretical mass of intact CFTR (168 kDa) in adenocarcinoma cell line, Calu-3, not in JCF-4. (C) The band detected by N-terminal epitope recognizing antibody (a) is the theoretical mass (60 kDa) of mutated CFTR elucidated from the sequence. The extra band (b) is the unique band in JCF-4. The band (c) is the theoretical mass of intact CFTR, which is the unique band in 253G1.

Supplementary Table 1, Exsome analysis revealed the homozygous 140del10 mutations in CFTR.

The detected variants were summarized. Note: two allele counts of del10 mutation was detected in genotypes.

Supplementary Table 2, List of antibodies used for this study.

The primary antibodies and the secondary antibodies are listed with information on antigen names involving epitope positions for CFTR, hosts, clonality, manufactures, ordering I.D.s, and dilutions for use.

Supplementary References

1. Nakagawa M, Koyanagi M, Tanabe K, et al. Generation of induced pluripotent stem cells without Myc from mouse and human fibroblasts. *Nat Biotechnol*. 2008;26(1):101-106. doi:10.1038/nbt1374

2. Okita K, Yamakawa T, Matsumura Y, et al. An Efficient Nonviral Method to Generate Integration-Free Human-Induced Pluripotent Stem Cells from Cord Blood and Peripheral Blood Cells. *Stem Cells*. 2013;31(3):458-466. doi:10.1002/stem.1293

3. Generation of human iPSCs from cells of fibroblastic and epithelial origin by means of the oriP/EBNA-1 episomal reprogramming system - PubMed. Accessed May 24, 2024. https://pubmed.ncbi.nlm.nih.gov/26088261/

4. Sanz J, von Känel T, Schneider M, Steiner B, Schaller A, Gallati S. The CFTR frameshift mutation 3905insT and its effect at transcript and protein level. *Eur J Hum Genet*. 2010;18(2):212-217. doi:10.1038/ejhg.2009.140

5. Sharma N, Evans TA, Pellicore MJ, et al. Capitalizing on the heterogeneous effects of CFTR nonsense and frameshift variants to inform therapeutic strategy for cystic fibrosis. *PLOS Genetics*. 2018;14(11):e1007723. doi:10.1371/journal.pgen.1007723

6. Wong AP, Bear CE, Chin S, et al. Directed differentiation of human pluripotent stem cells into mature airway epithelia expressing functional CFTR protein. *Nat Biotechnol*. 2012;30(9):876-882. doi:10.1038/nbt.2328

7. Ngan SY, Quach H, Dierolf J, et al. Modeling lung cell development using human pluripotent stem cells. Published online December 14, 2021:2021.07.16.452691. doi:10.1101/2021.07.16.452691

8. Prota LFM, Cebotaru L, Cheng J, et al. Dexamethasone Regulates CFTR Expression in Calu-3 Cells with the Involvement of Chaperones HSP70 and HSP90. *PLOS ONE*. 2012;7(12):e47405. doi:10.1371/journal.pone.0047405

9. Anoctamin 6 Contributes to Cl- Secretion in Accessory Cholera Enterotoxin (Ace)-stimulated Diarrhea: AN ESSENTIAL ROLE FOR PHOSPHATIDYLINOSITOL 4,5-BISPHOSPHATE (PIP2) SIGNALING IN CHOLERA - PubMed. Accessed May 24, 2024. https://pubmed.ncbi.nlm.nih.gov/27799301/

10. Kanda Y. Investigation of the freely available easy-to-use software “EZR” for medical statistics. *Bone Marrow Transplant*. 2013;48(3):452-458. doi:10.1038/bmt.2012.244
